# First, do no harm: impact of the transition to an integrated curriculum on medical knowledge acquisition of the transitional cohort

**DOI:** 10.1080/10872981.2021.2007561

**Published:** 2021-11-23

**Authors:** Kirstin Nackers, Raquel Tatar, Eileen Cowan, Laura Zakowski, Katharina Stewart, Sarah Ahrens, Laura Jacques, Shobhina Chheda

**Affiliations:** a Department of Pediatrics, University of Wisconsin School of Medicine and Public Health, Madison, WI, USA; b Healthy Minds Innovations, Madison, WI, USA; c Department of Medicine, University of Wisconsin School of Medicine and Public Health, Madison, WI, USA; d Department of Obstetrics and Gynecology, University of Wisconsin School of Medicine and Public Health, Madison, WI, USA

**Keywords:** Medical schools, clinical clerkship, program evaluation, medical education, national board of medical examiners, curriculum redesign, integrated clerkships, medical knowledge acquisition, subject exam

## Abstract

**Introduction:**

Many medical schools are moving toward integrated curricula in response to the 2010 Carnegie report. However, there is often apprehension that student performance on standard assessment metrics of medical knowledge acquisition could suffer during the transition period. Therefore, we sought to analyze the impact of curriculum redesign on the medical knowledge acquisition of the transitional cohort, as measured by NBME subject exam scores.

**Methods:**

The University of Wisconsin School of Medicine and Public Health Legacy curriculum followed a standard 2 + 2 medical school educational model, including traditional, department-based, third-year clinical clerkships. In the new ForWard curriculum, students enter clinical rotations one semester earlier, and those core clinical experiences are organized within four integrated blocks combining traditional clerkship specialties. This retrospective program evaluation compares NBME subject exam scores between the final cohort of Legacy third-year students (2016–17) and first cohort of ForWard students (2018) for the Adult Ambulatory Medicine, Medicine, Neurology, Obstetrics and Gynecology, Pediatrics, Psychiatry, and Surgery exams.

**Results:**

NBME subject exam mean scores ranged from 75.5–79.4 for the Legacy cohort and 74.9–78.7 for the ForWard cohort, with no statistically significant differences in scores identified for each individual exam analyzed. Results remained constant when controlled for student demographic variables.

**Discussion:**

Faculty and students may worry about impacts to the transitional cohort during curriculum redesign, however our results suggest no substantive negative effects to acquisition of medical knowledge during transition to an integrated curriculum. Further monitoring is necessary to examine whether medical knowledge acquisition remains stable or changes after the integrated curriculum is established.

## Introduction

In 2011, the University of Wisconsin School of Medicine and Public Health (UWSMPH) embarked on a curriculum redesign following recommendations from the 2010 Carnegie Foundation report, which encouraged medical schools to move toward a more integrated curriculum and away from traditional medical education [1,2]. Vertical integration, specifically, aligns well with the goals of several learning theories, including adult learning and motivation theories to enhance learning [3]. The new curriculum, named the ForWard curriculum, features an integrated design of the required preclinical and core clinical coursework and replaced a traditional 2 + 2 curriculum, referred to as the Legacy curriculum [4].

Recently, many medical schools have similarly planned or implemented new curricula [1,2]. As schools undergo curricular changes, there is often apprehension that student performance on standard assessment metrics related to medical knowledge acquisition might suffer during the transitional period. However, little has been published focusing on this specific time in curriculum redesign, to ensure transitional cohorts are performing adequately and are not inadvertently disadvantaged.

Published data on outcomes of new curricula once established demonstrate either no change or an improvement in NBME subject exam scores [5–7]. For example, The University of South Dakota Sanford School of Medicine offers the Yankton Ambulatory Program (YAP) to a subset of students who have the opportunity to complete all traditional M3 specialties as a yearlong longitudinal integrated clerkship in a multidisciplinary ambulatory setting. No significant differences were found when comparing the results of the Obstetrics and Gynecology, Internal Medicine, and Surgery NBME subject exams for 21 students in YAP and 43 students in a standard curriculum [5] Similarly, there was no difference in the Obstetrics and Gynecology, Pediatrics, Psychiatry, and Surgery NBME exams for 11 students in a control group of traditional clerkships and eight students (M3) in the pilot year of an integrated curriculum in the Harvard Medical School-Cambridge Integrated Clerkship [6,8] After three years, a subsequent study compared 27 students in this integrated curriculum with 45 students from a comparison cohort. They found no differences in the Obstetrics and Gynecology and Surgery NBME scores, but there was a statistically significant increase in the Pediatrics and Psychiatry NBME scores [7]

Although these studies are encouraging in that they do not show statistically significant decreases in NBME exam scores, and occasionally even show an increase, these studies were small and represented programs that provided an integrated curriculum only in the clinical years and only to a small subset of students at each institution. Further, none of these studies focus on the impact of the transitional period itself. It is important for individual institutions to understand how well students learn during the changeover period to ensure equitable education. Yet, to our knowledge, there are no published articles on student acquisition of medical knowledge as measured by NBME scores during the transitional year specifically, nor of an entire class-year cohort in a fully integrated curriculum spanning all four years of medical school.

Therefore, we evaluated NBME subject exam scores between the 2016–2017 Legacy third-year students and the 2018 first cohort of ForWard students for the Adult Ambulatory Medicine, Medicine, Neurology, Obstetrics and Gynecology, Pediatrics, Psychiatry, and Surgery exams. Standardized exams such as the National Board of Medical Examiners (NBME) Subject exams are standardized assessments of clinical medical knowledge and have been used in both the UWSMPH Legacy and ForWard curricula, allowing comparison of medical knowledge acquisition between the two cohorts.

## Methods

### Curricula integration changes

**Figure 1.**
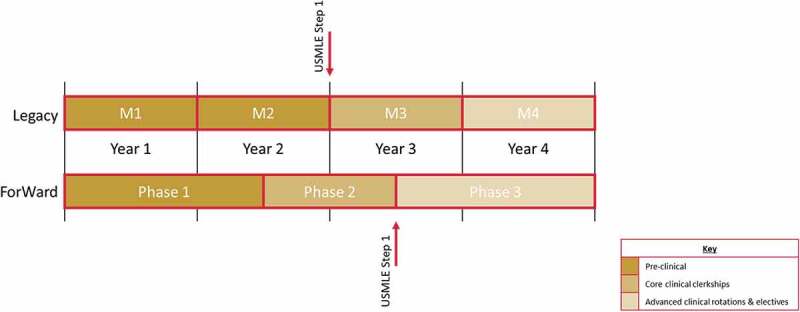
The University of Wisconsin School of Medicine and Public Health Legacy and ForWard curricula

Students matriculating into the UWSMPH medical doctorate program prior to the Fall 2016 semester followed the traditional educational model (Legacy curriculum): two years of preclinical education and two years of clinical education (Figure 1). Within this Legacy curriculum, students would take 4–6 weeks to prepare for and take USMLE Step 1 before entering third-year clerkships. The Legacy third year (M3) curriculum included seven required clinical clerkships that varied in length from four to eight weeks: Primary Care, Internal Medicine, Neuroscience, Obstetrics and Gynecology, Pediatrics, Psychiatry, and Surgery. The clerkships were located in Madison, Wisconsin and at several affiliate health care systems across the state with University of Wisconsin adjunct clinical faculty. Students concluded each clerkship with an NBME subject exam that contributed 25%-40% of the final clerkship grade (Table 1)

**Table 1.** Legacy M3 clerkships and forward phase 2 integrated blocks

	Clerkship	Duration	NBME Subject Exam
Legacy 2016–2017	Primary Care ^a^ (M3)	8 weeks	Adult Ambulatory Medicine
	Internal Medicine (M3)	8 weeks	Medicine
	Neuroscience (M3 or M4)	4 weeks	Neurology
	Obstetrics and Gynecology (M3)	6 weeks	Obstetrics and Gynecology
	Pediatrics (M3)	6 weeks	Pediatrics
	Psychiatry (M3)	4 weeks	Psychiatry
	Surgery (M3)	8 weeks	Surgery
	Anesthesiology (M3 or M4)	2 weeks	n/a
ForWard 2018	Acute Care Internal Medicine (8 weeks) Neurology (2 weeks) Psychiatry (2 weeks)	12 weeks	Medicine Neurology ^b^
	Chronic and Preventive Care Primary Care ^a^ (8 weeks) Neurology (2 weeks) Psychiatry (2 weeks)	12 weeks	Psychiatry ^b^ Adult Ambulatory Medicine
	Care Across the Life Cycle Pediatrics (4–6 weeks) Obstetrics and Gynecology (4–6 weeks) Geriatrics (2 weeks)	12 weeks	Obstetrics and Gynecology Pediatrics
	Surgical and Procedural Care Surgery (10 weeks) Anesthesiology (2 weeks)	12 weeks	Surgery

^a^The Primary Care experiences include Family Medicine, General Internal Medicine, and occasionally General Pediatrics.

^b^All students must complete either the standardized didactic curriculum or 4 weeks of clinical experience in Psychiatry and Neurology before taking the related exam.

**Figure 2.**
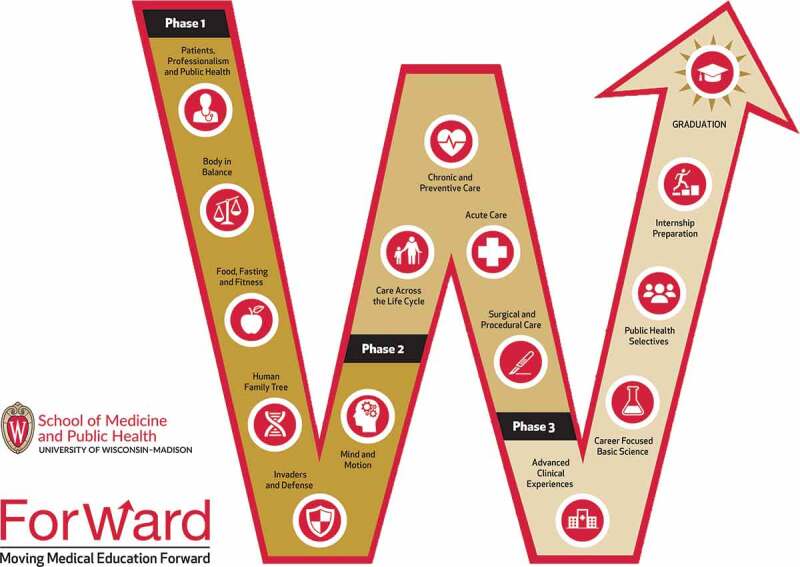
The University of Wisconsin School of Medicine and Public Health ForWard curriculum, detailed

A new integrated curriculum, called the ForWard curriculum, was implemented in 2016 [4] (Figures 1 and 2). The four-year ForWard curriculum has three phases and integrates fundamental sciences and clinical sciences. The curriculum also integrates ten critical content areas, referred to as threads. These threads include Ethics, Evidence-based medicine, Health information technology, Interpersonal and communication skills, Patient care, Professionalism and lifelong learning, Public Health, Quality improvement and patient safety, and Scientific inquiry. ForWard students complete only 3 semesters of pre-clinical education (Phase 1), then 2 semesters of required, integrated clinical blocks (Phase 2, analogous to clerkships during a traditional M3 year), concluding with three semesters of advanced clinical rotations and educational experiences in public health and basic science courses (Phase 3, analogous to a tradition M4 year). As in M3 year of the Legacy Curriculum, students in ForWard Phase 2 integrated blocks take place across the statewide campus. The four blocks are: Acute Care (Internal Medicine, Emergency Medicine, Neurology, and Psychiatry); Chronic and Preventive Care (Family Medicine, General Internal Medicine, Neurology, and Psychiatry); Care Across the Life Cycle (Obstetrics and Gynecology, Pediatrics and Geriatrics); and Surgical and Procedural Care (Surgery, Anesthesia, and other procedural specialties). Fundamental science and thread learning objectives are purposefully incorporated within each block. The same seven NBME subject exams are required to pass the associated block (Table 1). Following Phase 2, students take 4–6 weeks to prepare and take the United States Medical Licensing Examination (USMLE) Step 1 before advancing to Phase 3 of the curriculum.

### Standardized assessment data

We monitored outcomes of standardized NBME subject exams to assess the impact of the transition to the new curriculum on medical knowledge acquisition. In the final year (2016–2017) of the traditional Legacy curriculum, 183 M3 students completed at least one required M3 clinical clerkship. In the ForWard curriculum Phase 2 implementation year (2018), 162 students completed at least one integrated block. The 2017–2018 Legacy cohort was not included in the analysis because their clerkships were altered to account for the one-semester overlap with ForWard Phase 2 curricula in Spring 2018.

### Statistical analysis plan

NBME subject exam scores were compared using analysis of variance with covariates (ANCOVA), adjusting for student age, gender, extended status, and location of clerkship (Madison versus all other clinical sites). A student was designated as extended status if they required greater than the scheduled four semesters (Legacy) or three semesters (ForWard) to complete preclinical curriculum and would therefore require greater than four years to earn their MD. Reasons for extended status include leaves of absence, course failure, or enrollment in dual-degree programs completed prior to entering clinical rotations. We used SAS software, version 9.4, (SAS Institute Inc., Cary North Carolina) to conduct all statistical analyses.

Through the University of Wisconsin pre-Institutional Review Board process, this was deemed program evaluation and exempt from further IRB review.

## Results

The two study groups are described in Table 2; there were two statistically significant differences in the demographics of the study groups. ForWard students entering the clinical phase of training were younger (p < 0.001) and they were also less likely to have extended their medical training prior to starting the core clinical blocks (p < 0.001) compared to Legacy students entering their M3 year. 87% of Legacy students and 97% of ForWard students completed all subject exams during the study years. The median [interquartile range] of exams not completed was 1 [1–2] and 2 [1.75–2.5] for Legacy and ForWard, respectively. All exam scores were included in the analysis, including for students who did not complete all exams in the study period. NBME subject exam mean scores ranged from 75.5–79.4 for the Legacy cohort and 74.9–78.7 for the ForWard cohort (Table 3). When analyzing Legacy and ForWard students’ performances, we found no statistically significant differences in the mean NBME subject exam scores between the two cohorts, even when controlled for demographic variables.

**Table 2.** Demographics of legacy & forward cohorts

	Legacy n = 183	ForWard n = 162	p value
Age (mean [SD])	26.9 (3.2)	25.6 (2.4)	< 0.001
Female (n [%])	91 (50%)	80 (49%)	0.95
Race/ethnicity (n [%])			0.07
Non-Hispanic White	130 (71%)	98 (60%)	
Non-Hispanic Black	6 (3%)	17 (10%)	
Hispanic	5 (3%)	7 (4%)	
Asian	28 (15%)	23 (14%)	
Native American	3 (2%)	2 (1%)	
Other	11 (6%)	15 (9%)	
Extended preclinical enrollment	26 (14%)	5 (3%)	< 0.001

Abbreviations: SD indicates standard deviation.

**Table 3.** NBME subject exam scores; adjusted means (95% confidence intervals)

	Legacy		ForWard		
	n = 183		n = 162		
	Mean ^a^	95% CI	Mean ^a^	95% CI	Difference (95% CI)
Adult Ambulatory Medicine	75.8	74.4–77.2	75.4	73.8–77.1	−0.35 (−1.9–1.2)
Medicine	75.5	73.8–77.3	74.9	72.7–77.0	−0.65 (−2.6–1.3)
Neurology	77.1	75.3–78.9	77.7	75.7–79.6	0.52 (−1.31–2.36)
OB/GYN	79.4	77.9–80.9	78.7	76.9–80.4	−0.75 (−2.33–0.84)
Pediatrics	76.2	74.6–77.8	75.7	73.9–77.6	−0.48 (−2.17–1.21)
Psychiatry	75.9	74.5–77.3	75.5	73.8–77.1	−0.44 (−1.97–1.10)
Surgery	75.5	74.0–77.1	75.4	73.7–77.1	−0.13 (−1.67–1.40)

Abbreviations: CI indicates confidence interval.

^a^The analysis was adjusted by age, gender, extended status, and location (Madison vs. all other locations).

## Discussion

These results suggest that students in the transitional cohort acquired the same level of medical knowledge as in previous years, which is encouraging. Educators who have undergone a curriculum redesign can speak to the challenges of implementation, including ‘overlapping’ cohorts in core clinical rotations when the new curriculum includes earlier entry into clinical rotations [9]. Schools embark on curriculum redesign to improve education but may worry about impact on early cohorts before the new curriculum is well-honed. As such, it is essential to track assessment outcomes during periods of curricular change, not only to monitor students’ performance, but also to evaluate whether these changes are having an unanticipated impact on the cohort of students in the transition. The UWSMPH curriculum redesign included teaching across clinical departments, reinforcing fundamental science in the clinical blocks, and including content to create explicit curriculum related to non-medical knowledge competencies. These results indicate, despite these bold changes, that the first cohort of ForWard students maintained comparable medical knowledge acquisition compared to Legacy students.

Stable performance during the transition is very reassuring, though should not be misinterpreted as a comparison or evaluation of the new curriculum itself. Such an evaluation should compare cohorts spanning multiple years from both curricula, and consider additional outcomes beyond just student medical knowledge acquisition. To our knowledge, however, this analysis is the first to describe medical knowledge outcomes from an integrated curriculum for an entire medical school cohort (rather than just a subset of students) and for all the subject exams representative of the entire core clinical curriculum (as opposed to a select few specialties).

Although an intentional choice, one limitation of this study is that it represents just the first cohort of students in the ForWard curriculum. There is a possibility that students in a new curriculum may increase their individual study to ensure their personal exam scores are not at risk, although we have no evidence to suggest this is the case. On the other hand, it is also possible that students would study less for the exams that are weighted less in the overall assessment schema of the ForWard curriculum. A survey of students may have been beneficial to evaluate study patterns between the Legacy and ForWard cohorts, although unfortunately we did not have these data collected during the transitional year. Schools planning their own curriculum redesign could consider including a short survey to their transitional students to collect this information. Additionally, students participating in the first semester of the ForWard curriculum forged the path into the transition, overlapping in the core clinical environments with Legacy students. This may have impacted their clinical experiences, though the impact of the overlap on student clinical schedules was not quantified. Regardless, this is a critical period to analyze because it is a common concern faced by medical schools redesigning curricula with earlier entry to clinical clerkships.

In conclusion, there were no statistically significant differences in NBME scores between students in the transitional year of a curriculum with integrated preclinical and clerkship years versus a traditional non-integrated educational model with departmental clerkships at our large medical school. These results indicate there are no substantive negative effects to student acquisition of medical knowledge during the integrated curriculum transition period. This information is critical given that 65% of medical schools are currently transitioning or planning to transition to new curricula[1]. Continued monitoring of data is necessary.
